# Growth Characterization of Intermetallic Compound at the Ti/Al Solid State Interface

**DOI:** 10.3390/ma12030472

**Published:** 2019-02-04

**Authors:** Yangyang Zhao, Jiuyong Li, Ranfeng Qiu, Hongxin Shi

**Affiliations:** 1 School of Materials Science and Engineering, Henan University of Science and Technology, Luoyang 471039, China; zhaoyy03@163.com (Y.Z.); 15737936609@163.com (J.L.); 2 Henan Key Laboratory of Advanced Non-ferrous Metals, Luoyang 471003, China; 3 Collaborative Innovation Center of Nonferrous Metals, Luoyang 471039, China

**Keywords:** Titanium, Aluminum, growth kinetic, TiAl_3_

## Abstract

Ti-Al diffusion couples, prepared by resistance spot welding, were annealed up to 112 h at 823, 848, and 873 K in ambient atmosphere. The interfacial microstructure was observed and analyzed using SEM and TEM. The growth characterization of intermetallic compound formed at the Ti/Al solid state interface was investigated. Only TiAl_3_ phase was detected in the interfacial zone, and its growth was governed by reaction-controlled mechanism in the previous period and by diffusion-controlled mechanism in the latter period. The activation energies were 198,019 and 122,770 J/mol for reaction-controlled and diffusion-controlled mechanism, respectively.

## 1. Introduction

Titanium and aluminum are attractive engineering metals in industrial applications because of their excellent properties, therefore, achieving the joining between two kinds of materials not only can combine their excellent characteristics, reduce the weight of the structure, but also save costs and further expand their application prospects. In view of this, some welding methods such as friction stir welding [1,2], roll-bonding [3], explosive welding [4], ultrasonic welding [5], resistance spot welding [6], laser welding [7] and arc welding-brazing [8,9] were applied to join titanium and aluminum. Previous studies reveal that the brittle intermetallic compounds (IMCs) layer formed at the welding interface seriously impair the mechanical properties of the joint [10,11]. On the other hand, Ti-TiAl_3_ metal-intermetallic laminate (MIL) composites are considered as a great potential material for aerospace, automotive, and other structural applications because of its combination of high strength, toughness, and stiffness at a lower density than monolithic titanium or other laminate systems [12]. To fabricate the MIL composites, aluminum and titanium sheets are pressed together by use of explosive welding [12,13,14] and hot press bonding [15,16,17,18]. During producing of MIL, it is indispensable to form a continuous IMCs layer at Al/Ti interface, so much so that a post-bonding annealing treatment is often employed [12,13,14,15,16,17]. Therefore, it is necessary to understand the growth characteristics of IMCs layer in both cases of Al/Ti welding and MIL production so as to control its growth.

In recent years, the growth kinetics of Ti-Al IMCs, especially in solid state, has attracted wide attention [12,13,14,15,16,17,18,19,20,21,22,23]. In general, the thickness of interfacial IMCs layer depends on growth time (*t*), kinetic exponent (*n*) and growth rate constant (*k*) for certain temperature [19,21,23]. According to the value of kinetic exponent, the IMCs growth mechanism can be divided into reaction-controlled and diffusion-controlled [13]. In works of Mirjalili et al., Ti/Al diffusion couples were annealed in time range from 0 to 96 h at 823, 848, 873, 898, 913 and 923 K, and it was demonstrated that diffusion-controlled mechanism was the only one which is present during TiAl_3_ formation in the whole process [19,20]. Xu et al. also studied the growth kinetics of IMCs formed at solid-state Ti/Al interface, and declared that the values of the kinetic exponent at the temperatures of 793, 823, 848, 873, 903 and 923 K were 0.55, 0.49, 0.47, 0.83, 1.08 and 1.06, respectively [21]. They divided the kinetic exponent *n* into two groups (*n* ≤ 0.5 and *n* > 0.5), and claimed that the IMCs growth was governed by diffusion-controlled mechanism at the low temperature and by reaction-controlled mechanism at the high temperature [21]. Fronczek et al. investigated the interface of explosively welded Ti/Al samples annealed for various time at the temperature of 773, 825 and 903 K, and confirmed that the growth of IMCs was controlled by reaction, mixed mechanism of both reaction and diffusion, and diffusion for 1.5~5, 5~36, 36~100 h annealing time at certain temperature, respectively [22,23]. Farzad et al. concluded that the IMCs growth was governed by reaction-controlled mechanism in the previous period and by diffusion-controlled mechanism in the latter period for certain temperature; and that the reaction-controlled stage broadened by decreasing the temperature via investigating explosively welded Ti/Al joint annealed for up to 260 h at 903, 873, and 843 K [12]. From the above, there are some discrepancies about the mechanism of IMCs growth kinetics at Ti/Al interface. Therefore, further studies are necessary on growth kinetics of Ti-Al IMCs at solid-state interface.

In the present work, Ti/Al diffusion couples were prepared by resistance spot welding and employed to clarify the growth mechanism of Ti-Al IMCs. The growth rate constant and activation energy for interfacial IMCs formation were calculated; kinetic exponent for interfacial IMCs growth was discussed emphatically. Finally, a growth kinetics model was established to predict the growth thickness of the IMCs at Ti-Al solid-state interface.

## 2. Experimental Procedure

Commercially pure titanium (TA2, henceforth calls Ti) with dimensions of 30 × 30 × 1 mm and aluminum (1050A, henceforth calls Al) with dimensions of 30 × 30 × 2 mm were employed as the base materials. Their chemical compositions are listed in Table 1. Ti-Al diffusion couples were prepared by resistance spot welding to study the growth mechanism of Ti-Al IMCs. Prior to welding, the base materials were ground by abrasive paper and degreased by use of acetone. A titanium sheet and an aluminum sheet were overlapped for welding. As diffusion couples, the Ti-Al interface should be well bonded, and little IMCs are formed at the interface. Hence, smaller heat output is required during resistance spot welding process. In the study, the welding parameters were determined as welding current of 10 kA, welding time of 200 ms and electrode force of 1.7 kN.

The welded specimens were annealed up to 112 h at three different temperatures: 823, 848, and 873 K in ambient atmosphere. After annealing, the samples were cooled in air and prepared for metallographic examination by sectioning transverse to the reaction interface. The specimens were mounted in epoxy resin, then ground with abrasive paper up to 2000 grits and polished to obtain mirror-finished surfaces for microstructure observation and chemical composition analysis. The interfacial microstructure of the samples were investigated by using a transmission electron microscope (TEM, JEM-2100, JEOL, Tokyo, Japan; acceleration voltage: 200 kV) and a scanning electron microscope (SEM, JSM-6300, JEOL, Tokyo, Japan) equipped with energy dispersive X-ray spectroscopy (EDX, EDAX, Phoenix, USA). The thickness of the reaction layers at Ti/Al interfaces was measured from scanning electron microscope micrographs of the cross-sections. An X-ray diffractometer (XRD, Bruker, Karlsruhe, Germany) was used to identify the phases of the samples.

## 3. Results

### 3.1. Morphological Characterization and Phase Identification

**Figure 1:**
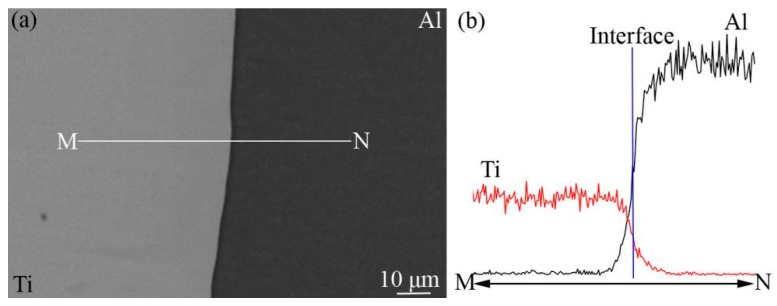
Typical SEM images after welding (**a**) and EDX results (**b**).

Figure 1a shows the typical SEM image of Ti-Al initial interface after welding. The interface was fairly flat and no defects such as voids and cracks were found in the interface region, which indicate that Ti and Al were boned well. It is pretty obvious that IMCs can not be observed at the Ti-Al initial interface. Figure 1b shows EDX results detected from M to N. As can be seen from the curves, there is a distinct element diffusion region near the Ti-Al interface.

**Figure 2:**
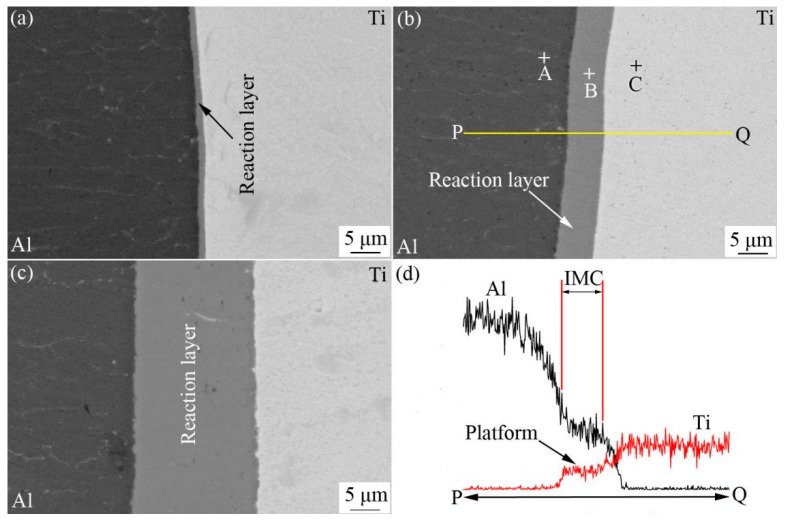
SEM images of the interfacial zone of samples annealed and EDX results; (**a**) annealing time 5 h, (**b**) annealing time 32 h, (**c**) annealing time 96 h, (**d**) EDX results.

Figure 2a–c show SEM images of Ti-Al interface annealed at 823 K for annealing time of 5, 32 and 96 h, respectively. It can be seen that a deep gray reaction layer formed between Ti and Al, and the thickness of the reaction layer increased remarkably with the increase of the annealing time. As shown, the interfaces between the reaction layer and the base materials were slightly wavy, and the shapes of the waves become larger with the increase of annealing time, which means that the growth of the reaction layer is not absolutely homogeneous. Figure 2d shows the results of EDX along line PQ (see Figure 2b). It can be seen from the composition distribution curves that there is a platform at the interfacial zone. This means that the composition of reaction layer is stable and it is an intermetallic compound (IMC) layer.

The quantitative analysis results by EDX at the positions of A, B and C shown in Figure 2b are listed in Table 2. As shown, a rough composition of the reaction layer (position of B) is of 75.12 at.% Al and 24.84 at.% Ti which corresponds to TiAl_3_. Therefore, the reaction layer formed at the interfacial zone was identified as TiAl_3_ layer. In addition, Ti and Al were also detected in Al (position of A) and Ti (position of C) side near the interface, respectively. This reveals that Ti and Al atoms diffused through the TiAl_3_ layer to Al and Ti side, respectively.

**Figure 3:**
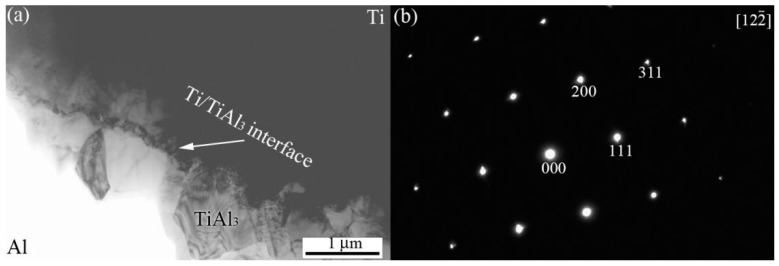
Interfacial zone TEM images of the Ti-Al sample annealed for 1 h at 873 K (**a**) and the electron diffraction patterns taken form the IMC layer (**b**).

Figure 3a shows the typical bright field image of interfacial zone taken from the Ti/Al sample annealed for 1 h at 873 K. In this image, an approximately 1 µm thick layer structure was observed. Figure 3b shows the selected area electron diffraction patterns of interfacial IMC layer. According to the analyses of selected area electron diffraction patterns, it was identified that the interfacial IMC was TiAl_3_.

**Figure 4:**
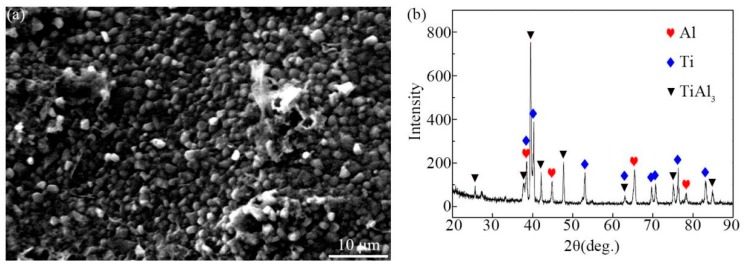
Fracture morphology of samples annealed for 4 h at 873 K (**a**) and XRD pattern taken from the fracture surface (**b**).

Figure 4a shows the TiAl_3_ side fracture surface morphology of the Ti/Al sample annealed for 4 h at 873 K, from which residual aluminum was removed by use of mechanical methods to expose TiAl_3_. Some particles with a diameter of approximately 1 μm were observed. Figure 4b shows X-ray diffraction pattern of the fracture surface. Only TiAl_3_ phase was detected except for Ti and Al. This also verifies that only TiAl_3_ formed at the interface during annealing.

### 3.2. Intermetallic Layer Thickening and the Kinetics of TiAl_3_ Formation

**Figure 5:**
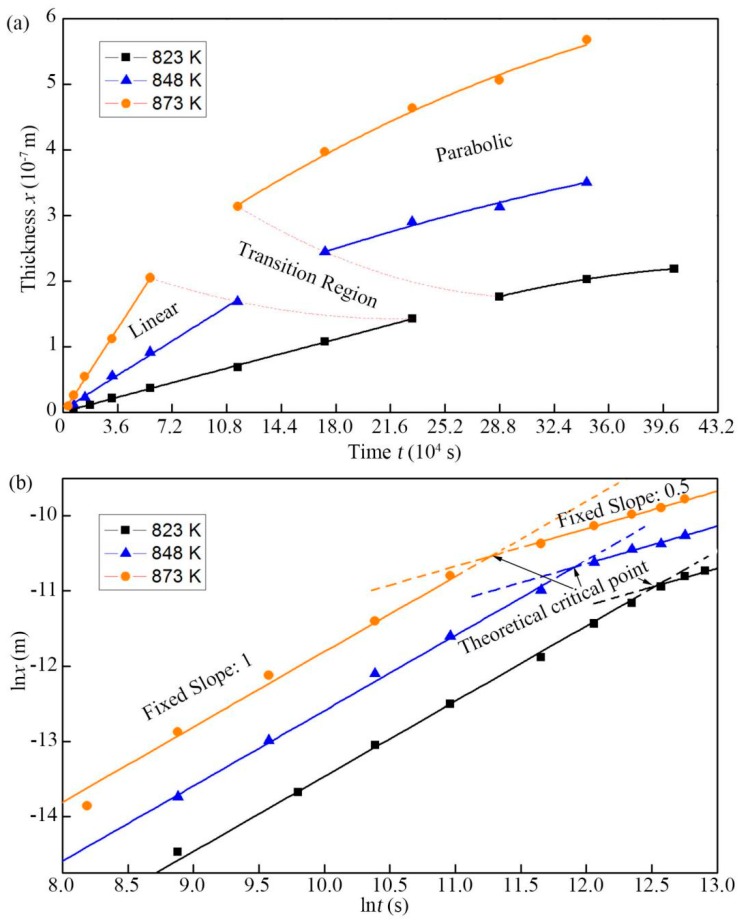
The average thickness of TiAl_3_ layer at different experiment conditions in normal scale (**a**) and logarithmic scale (**b**).

The average thickness of TiAl_3_ layer at different experiment conditions were measured. The layer thickness variation as a function of annealing time in normal scale is shown in Figure 5a. Two stages depending on the relationship between the thickness of TiAl_3_ layer and annealing time at three temperatures can be seen in Figure 5a, where the linear and parabolic stage represent that the layer thickness increases with time in the form of straight line and parabola, respectively. Obviously, as the temperature increases, the linear stage became shorter and shorter. In addition, there is a transition region between the linear and parabolic stage under the condition of parameters used in this study.

As well known, the thickness of interfacial IMC layer is a power function of time. Therefore, the TiAl_3_ layer thickness at each temperature can be described by the following equations:

*x* = *kt^n^* (1)

ln *x* = *n*ln*t* + ln*k* (2)

in which *x*, *k*, *t*, and *n* are the thickness of the TiAl_3_ layer (m), growth rate constant (m/s^n^), annealing time (s), and kinetic exponent, respectively.

The layer thickness variation as a function of annealing time in logarithmic scale is shown in Figure 5b. Taking into account the experimental errors, the present data were fitted a line. The slope and intercept of each line represent its *n* and ln*k* value. These values are collected in Table 3. As mentioned above, the IMC layer growth mechanism is divided into reaction-controlled (*n* value is 1) and diffusion-controlled (*n* value is 0.5) mechanism. As shown in Figure 5b, TiAl_3_ layer growth was governed by reaction-controlled mechanism in the previous period and by diffusion-controlled mechanism in the latter period for each annealing temperature in this study; and the reaction-controlled stage broadened by decreasing the temperature. This is consistent with the results reported by Farzad et al. [12].

The growth rate constant *k* can be expressed as an Arrhenius function as follows:

*k* = *k*_0_exp(−*Q*/*RT*) (3)

ln *k* = ln *k*_0_ − *Q*/*RT* (4)

where *k*_0_ is a temperature-independent constant, *T* is the absolute temperature (K), *R* is the gas constant, and *Q* is the activation energy for TiAl_3_ growth (J/mol).

**Figure 6:**
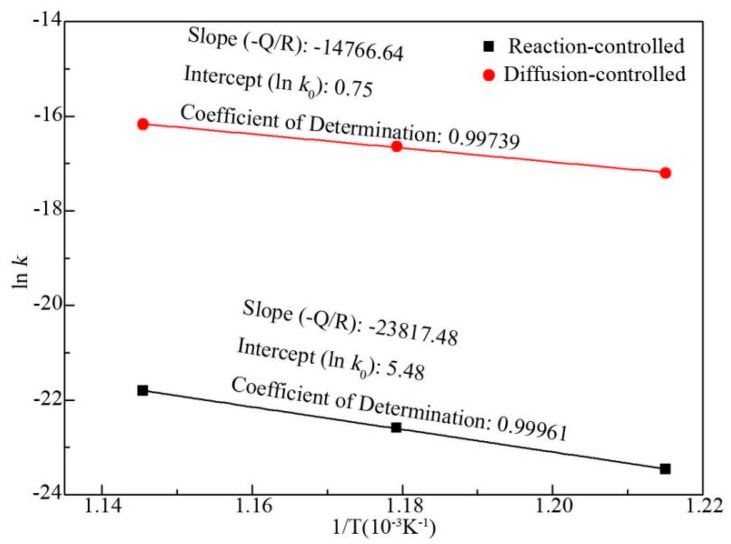
ln*k* versus the reciprocal temperature for two different growth mechanisms.

According to Equation (4), ln*k* is plotted versus reciprocal temperature in Figure 6. The slope of line represents the value of −*Q*/*R*. Accordingly, the activation energies of *Q_r_* (reaction-controlled mechanism) and *Q_d_* (diffusion-controlled mechanism) for TiAl_3_ growth can be calculated. They were 198,019 and 122,770 J/mol, respectively.

Moreover, the corresponding values of *k_0r_* and *k_0d_* were calculated to be 240.11 and 2.13 m/s, respectively. It should be mentioned that the subscripts *r* and *d* at *Q* and *k*_0_ represent the reaction-controlled and diffusion-controlled mechanism, respectively. Finally, growth kinetics model based on two growth mechanisms were described by two equations:

*x_r_* = 240.11 exp(−198019/*RT*)*t*   (Reaction-controlled) (5)

*x_d_* = 2.13 exp(−122770/*RT*)*t*^0.5^   (Diffusion-controlled) (6)

where *x_r_* and *x_d_* are the thickness of TiAl_3_ layer governed by reaction-controlled and diffusion-controlled, respectively.

## 4. Discussion

### 4.1. Formation of TiAl_3_

In this study, only TiAl_3_ phase formed at the Ti-Al interface during annealing in the temperature range of 823–873 K, even though the Ti-Al binary phase diagram suggests other IMCs, such as Ti_2_Al_5_, TiAl_2_, TiAl and Ti_3_Al, should also be formed between Ti and Al [24]. This can be explained from both thermodynamics and diffusion kinetics. From thermodynamics side, TiAl_3_ has lowest Gibbs standard free energy of formation among Ti_3_Al, TiAl and it [25]. In comparison with TiAl_3_, the formation of TiAl_2_ or Ti_2_Al_5_ with lower Gibbs standard free energy starts with TiAl and goes through a series of chemical reactions, thus TiAl_2_ and Ti_2_Al_5_ can be ignored here. In terms of diffusion dynamics, TiAl_3_ has the most negative effective heat of formation (∆H^m^), and is expected to be the first phase to form in the diffusion zone among Ti-Al binary system compounds [21]. And so, only TiAl_3_ was detected at the interface of annealed Ti/Al sample in the study. This is in agreement with the results obtained in other studies [13,19,20,23].

It should be noted that there is no obvious incubation period of TiAl_3_ formation in Figure 5a. This is not in accordance with previous reports [12,20,23]. The incubation means a period of time for Ti and Al atoms diffusing with each other at the interface before the formation of TiAl_3_. However, the mutual solubility of Al and Ti is very small, so it is easy to reach saturation state. And a distinct element diffusion region at the Ti-Al interface has been formed during spot welding process (Figure 1), which greatly shortens the incubation period. Therefore, in this experiment, it is reasonable to believe that the incubation period of TiAl_3_ formation cannot affect the calculation of the growth kinetics and can be neglected.

### 4.2. Govern Mechanism Criterion of TiAl_3_ Layer Growth

**Figure 7:**
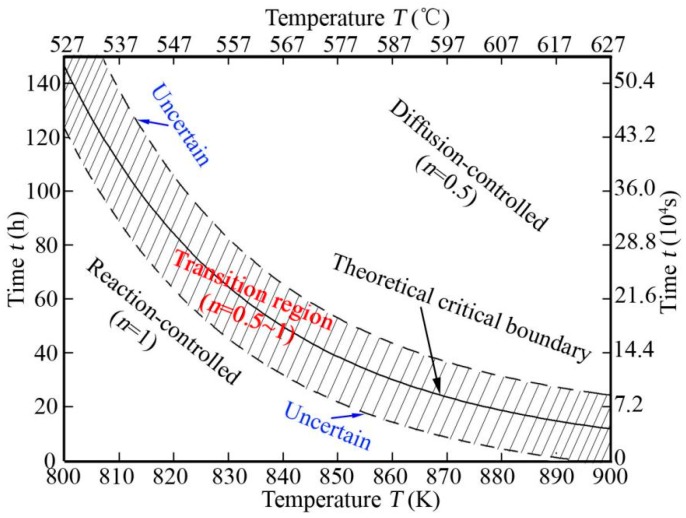
Time vs. critical temperature.

As mentioned above, derived Equations (5) and (6) are formulas for calculating the thickness of TiAl_3_ layer (*x*) in the case of both reaction-controlled and diffusion-controlled mechanism, respectively. However, this is not enough to predict TiAl_3_ layer thickness for certain annealing temperature and time, because it is unclear whether reaction-controlled and diffusion-controlled mechanism to be employed. Therefore, it is necessary to determine which govern mechanism to be used before predicting TiAl_3_ layer thickness. Unfortunately, there are few reports on this aspect so far. Based on this, a criterion for the govern mechanism of TiAl_3_ layer growth was put forward in this study. Theoretically, at the theoretical critical point shown in Figure 5b, the growth of TiAl_3_ layer can be seen as governed by either reaction-controlled mechanism or diffusion-controlled mechanism, which indicate that the value of *x_r_* and *x_d_* are equal. Then, a relationship between the critical time and temperature can be derived from Equations (5) and (6). The relationship was diagrammed as a theoretical critical boundary in Figure 7.

**Figure 8:**
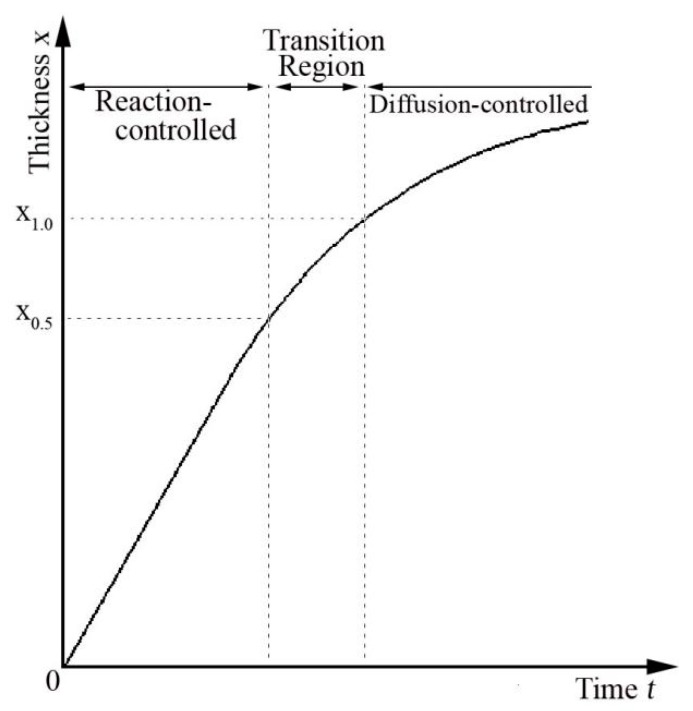
Schematic diagram of growth controlled mechanism.

In fact, there is a transition region between reaction-controlled and diffusion-controlled mechanism as shown in Figure 5a, rather than a definite line. This was also reported as a mixed mechanism of both reaction-controlled and diffusion-controlled mechanism (*n* = 0.5~1.0) in other studies [12,21,23]. This can be qualitatively explained by use of theories reported by Dybkov as shown in Figure 8 [26]. The growth of TiAl_3_ is a result of reaction between diffusion atoms through IMC layer and base metals at both interfaces of Ti/TiAl_3_ and Al/TiAl_3_. In the previous period, the growth of TiAl_3_ layer are governed by reaction-controlled mechanism at both interfaces of Ti/TiAl_3_ and Al/TiAl_3_, which is attributed to that the diffusion of Al and Ti atoms across the TiAl_3_ layer is sufficient because the TiAl_3_ layer is thinner. With the thickening of TiAl_3_ layer, the growth of TiAl_3_ layer at an interface is governed by diffusion-controlled mechanism when its thickness reaches *X*_0.5_, whereas its growth is still controlled by reaction-controlled mechanism at the other interface. This is because the reaction between Al and Ti at the both interfaces are not synchronized and the diffusion rates of Al and Ti atoms across the TiAl_3_ layer are also different. In the transition region, the growth of total TiAl_3_ layer is governed by the mixing mechanism of both reaction-controlled and diffusion-controlled mechanism (*n* = 0.5~1.0). When the thickness of TiAl_3_ layer reaches *X*_1.0_, its growth is governed by diffusion-controlled mechanism at both interfaces, which is due to that the diffusion of Al and Ti atoms are not sufficient because the TiAl_3_ layer is thicker.

Therefore, there is a transition region between reaction-controlled and diffusion-controlled mechanism as shown in Figure 7. When the annealing time and temperature employed are in the transition region, the growth of TiAl_3_ layer is governed by the mixing mechanism. Its growth is governed by reaction-controlled and diffusion-controlled mechanism when the annealing time and temperature employed are under and above the transition region, respectively.

In the study, the growth kinetics model based on mixing mechanism, the boundaries between reaction-controlled and mixing mechanism, and between diffusion-controlled and mixing mechanism are not yet uncertain. This needs further study and will be reported separately.

### 4.3. Growth Process of TiAl_3_

**Figure 9:**
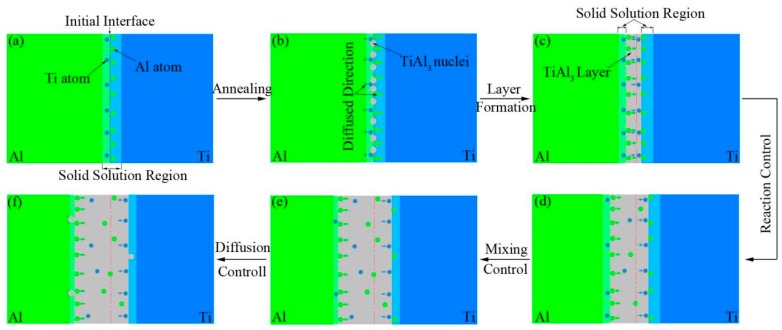
Schematic of the growth process of TiAl_3_ layer; (**a**) welding interface, (**b**) nucleation of TiAl_3_, (**c**) formation of continuous TiAl_3_ layer, (**d**) reaction -controlled, (**e**) mixing-controlled, (**f**) diffusion-controlled.

In summary, the growth process of TiAl_3_ layer at Ti/Al solid state interface is illustrated in Figure 9. During spot welding, Ti and Al atoms diffused with each other and formed a solid solution region of Ti(Al) and Al(Ti) respectively on both sides of the welding interface of Ti/Al as illustrated in Figure 9a. And then, the IMC of TiAl_3_ nucleated and grew during annealing process as illustrated Figure 9b–f.

In the initial stage of annealing, diffusion of Ti and Al atoms with each other caused that the solid solution of Ti(Al) and Al(Ti) reached saturation on both sides of the welding interface and TiAl_3_ nucleated at the part of the interface where would be higher energy as illustrated in Figure 9b. As the heating continues, TiAl_3_ also nucleated at another part of the interface because it is harder that Ti and Al atoms diffuse through formed TiAl_3_ in comparison with where TiAl_3_ was not formed. Whereupon, a thin continuous layer of TiAl_3_ formed at the welding interface as illustrated in Figure 9c.

After the formation of TiAl_3_ continuous layer, Ti and Al atoms diffused through the layer and reacted with each other, and then generated TiAl_3_ at the Ti/TiAl_3_ and the TiAl_3_/Al interfaces as illustrated in Figure 9d. Because the TiAl_3_ layer was thinner, the growth of TiAl_3_ layer was governed by reaction-controlled mechanism at both interfaces of Ti/TiAl_3_ and TiAl_3_/Al. With the growth of the TiAl_3_ layer, its growth was governed by mixing mechanism as illustrated Figure 9e. At the interface of Ti/TiAl_3_, the control mechanism of TiAl_3_ layer growth first changed from reaction-controlled to diffusion-controlled. This is because the diffusion of Al atoms is not sufficient for the reaction at the Ti/TiAl_3_ interface where need more Al atoms to react with Ti from the stoichiometry of the product phase in comparison with the interface of TiAl_3_/Al. When TiAl_3_ layer reached a certain thickness (*X*_1.0_), its growth was governed by diffusion-controlled mechanism after the control mechanism of TiAl_3_ layer growth also changed to diffusion-controlled at the interface of Ti/TiAl_3_ as illustrated in Figure 9f.

During growth of TiAl_3_ layer, one Ti atom needs three diffused Al atoms to react at the Ti/TiAl_3_ interface, whereas three Al atoms need one diffused Ti atom to react at the TiAl_3_/Al interface. From this respect, the growth rate in atom percent at the TiAl_3_/Al interface is nine times higher than the Ti/TiAl_3_ interface. Therefore, TiAl_3_ layer mainly grew toward the Al side, although diffusion Al atoms in TiAl_3_ layer is slight faster than that of Ti atom [27]. This is also consistent with the experimental results.

## 5. Conclusions

The growth mechanism of interfacial IMC layer formed in the Ti/Al diffusion couples has been investigated. The main conclusions are summarized as follows:

(1) Only TiAl_3_ phase was detected in the interfacial zone of Ti/Al sample annealed for up to 112 h at 823, 848, and 873 K.
(2) The growth of TiAl_3_ layer was governed by reaction-controlled mechanism in the previous period and by diffusion-controlled mechanism in the latter period for annealing temperature of 823, 848, and 873 K.
(3) The activation energies for reaction—controlled and diffusion-controlled mechanism were calculated to be 198,019 and 122,770 J/mol, respectively.
(4) A criterion for the govern mechanism of TiAl_3_ layer growth was put forward.

## Figures and Tables

**Table 1:** Chemical compositions of materials (Mass %).

Materials	C	N	O	H	Fe	Al	Ti	Si	Cu	Mn	Mg	Zn
TA2	0.008	0.005	0.041	0.0006	0.029	0.015	Bal.	-	-	-	-	-
1050A	-	-	-	-	0.4	Bal.	0.05	0.25	0.05	0.05	0.05	0.05

**Table 2:** Composition of points in Figure 2c by EDX.

Position	Composition (at. %)		Phase
	Al	Ti	
A	0.13	99.87	Ti
B	75.12	24.84	TiAl_3_
C	99.76	0.24	Al

**Table 3:** Values of ln*k* at different mechanisms for three temperatures.

Temperature/K	Fixed *n* (slope)	Mechanism	Ln*k* (Intercept)	*r* ^2^
823	1	Reaction controlled	−23.46	0.99748
823	0.5	Diffusion controlled	−17.20	0.99823
848	1	Reaction controlled	−22.59	0.99667
848	0.5	Diffusion controlled	−16.63	0.99910
873	1	Reaction controlled	−21.81	0.98717
873	0.5	Diffusion controlled	−16.17	0.99622
